# Out in the Cold: Identification of Genomic Regions Associated With Cold Tolerance in the Biocontrol Fungus *Clonostachys rosea* Through Genome-Wide Association Mapping

**DOI:** 10.3389/fmicb.2018.02844

**Published:** 2018-11-22

**Authors:** Martin Broberg, Mukesh Dubey, Man-Hong Sun, Katarina Ihrmark, Hans-Josef Schroers, Shi-Dong Li, Dan Funck Jensen, Mikael Brandström Durling, Magnus Karlsson

**Affiliations:** ^1^Department of Forest Mycology and Plant Pathology, Swedish University of Agricultural Sciences, Uppsala, Sweden; ^2^Key Laboratory of Integrated Pest Management in Crops, Ministry of Agriculture, Institute of Plant Protection, Chinese Academy of Agricultural Sciences, Beijing, China; ^3^Agricultural Institute of Slovenia, Ljubljana, Slovenia

**Keywords:** biocontrol, *Clonostachys rosea*, comparative genomics, genome sequencing, GWAS

## Abstract

There is an increasing importance for using biocontrol agents in combating plant diseases sustainably and in the long term. As large scale genomic sequencing becomes economically viable, the impact of single nucleotide polymorphisms (SNPs) on biocontrol-associated phenotypes can be easily studied across entire genomes of fungal populations. Here, we improved a previously reported genome assembly of the biocontrol fungus *Clonostachys rosea* strain IK726 using the PacBio sequencing platform, which resulted in a total genome size of 70.7 Mbp and 21,246 predicted genes. We further performed whole-genome re-sequencing of 52 additional *C. rosea* strains isolated globally using Illumina sequencing technology, in order to perform genome-wide association studies in conditions relevant for biocontrol activity. One such condition is the ability to grow at lower temperatures commonly encountered in cryic or frigid soils in temperate regions, as these will be prevalent for protecting growing crops in temperate climates. Growth rates at 10°C on potato dextrose agar of the 53 sequenced strains of *C. rosea* were measured and ranged between 0.066 and 0.413 mm/day. Performing a genome wide association study, a total of 1,478 SNP markers were significantly associated with the trait and located in 227 scaffolds, within or close to (< 1000 bp distance) 265 different genes. The predicted gene products included several chaperone proteins, membrane transporters, lipases, and proteins involved in chitin metabolism with possible roles in cold tolerance. The data reported in this study provides a foundation for future investigations into the genetic basis for cold tolerance in fungi, with important implications for biocontrol.

## Introduction

As the human global population grows and plant pathogens and pests increase and spread, sustainable agricultural production, and disease control increases in importance to ensure future food security (Syed Ab Rahman et al., 2018). Biological control agents (BCAs) demonstrate an environmentally sustainable and potent alternative to current industrial pesticide practices in agriculture, due to the risk chemicals may pose to human and animal health (Syed Ab Rahman et al., 2018). The mycoparasitic fungus *Clonostachys rosea* (Link: Fr.) Schroers, Samuels, Seifert & W. Gams, comb. nov. (Schroers et al., 1999) is a widely studied BCA, effective against several economically important fungal plant pathogens such as *Botrytis cinerea, Bipolaris sorokiniana, Fusarium* spp., and *Sclerotinia sclerotiorum* (Knudsen et al., 1995; Sutton et al., 1997; Teperi et al., 1998; Xue et al., 2009; Rodríguez et al., 2011). The sexual form (teleomorph) is described as *Bionectria ochroleuca* (Schw.) Schroers & Samuels, and certain strains readily produce sexual fruiting bodies (perithecia) in single culture indicating its homothallic mode of reproduction (Schroers et al., 1999). The biocontrol effect of *C. rosea* can be derived from its ability to directly parasitize plant pathogenic fungi, but also from exploitation competition for space and nutrients and from interference competition via antibiosis (Li et al., 2002; Rodríguez et al., 2011; Jensen et al., 2017).

Active growth in colder temperatures (below 15°C), is an important trait for a BCA to be effective in the field in temperate climates such as in the Nordic countries. This enables rapid colonization of seeds, wounds and other substrates for efficient exploitation competition, but is also important for maintaining high metabolic activity. This trait was originally one of the reasons for the selection of certain *C. rosea* strains as BCAs, e.g., for control of *F. culmorum* on wheat and barley at 15°C (Knudsen et al., 1995; Teperi et al., 1998). On the molecular level, mechanisms of cold tolerance in fungi partly overlap with other abiotic stress tolerances. Estimations suggest that approximately 85% of the biosphere is constantly subjected to temperatures of 5°C or lower (Li et al., 2012). Cold tolerance can be associated with changes in cell wall composition (carbohydrates, proteins and pigments), changes in lipid and osmolyte composition of cell membranes and the RNA translation and protein folding machinery (RNA helicases, trehalose, chaperone proteins), alcohol metabolism, transport proteins and mitochondria (Ellison et al., 2011; Blanc et al., 2012; Treseder and Lennon, 2015). Temperature such as cold has also been identified as a major environmental factor for organisms to adapt to, as it is one of the major environmental forces limiting life (D'Amico et al., 2002; Blanc et al., 2012; Li et al., 2012). Cold adaptation has been studied across free-living and plant-associated fungi to assess the temperature effects on adaptation, for example as a driving force for environmental colonization in plant-associated fungi and population divergence in fungi such as *Neurospora crassa* (Robinson, 2001; Ellison et al., 2011; Li et al., 2012). For fungi, cold tolerance may further be associated with osmotic stress tolerance, and spore survivability in low water, low nutrient conditions (Ruisi et al., 2007).

As genome sequencing has become cheaper and more user friendly, performing large-scale comparative genomics is now a standard practice for the identification of single nucleotide polymorphisms (SNPs), and determining their association with phenotypes of interest using genome-wide association studies (GWAS). The GWAS approach allows for statistical determination of SNPs of interests over a population of individuals with a shared evolutionary history. Previously, GWASs have been performed on fungal pathogens such as *Heterobasidion annosum, Zymoseptoria tritici, Parastagonospora nodorum*, and *Fusarium graminearum* (Dalman et al., 2013; Gao et al., 2016; Talas et al., 2016; Hartmann et al., 2017). However, as far as we are aware, no GWAS on fungal BCAs have been performed to date. The *C. rosea* strain IK726 genome was originally sequenced using short reads of the Illumina platform (Karlsson et al., 2015). However, the modern sequencing technology of the PacBio platform is allowing for more comprehensive and accurate genomics, by sequencing reads that are thousands to hundreds of thousands of base pairs (bp) in length instead of a few hundred bp (Roberts et al., 2013). This provides a very potent framework when combined with short read sequencing for assembling genomes (Schadt et al., 2010; Rhoads and Au, 2015; Chakraborty et al., 2016).

In this study, we evaluate the potential for performing GWAS in a fungal BCA. We present a re-sequenced genome of *C. rosea* IK726, combining the previously published genome scaffolds with long contigs gained from PacBio sequencing into a second, improved version of the genome, expanding the size from 58.3 to 70.7 Mbp and adding 6,497 predicted genes to a total of 21,246. In addition, we used Illumina technology to re-sequence the genomes of 52 additional *C. rosea* strains and performed a GWAS for growth rate in cold temperature (10°C). The analysis identified 1,478 SNP markers associated with the trait and certain genes located in the vicinity were predicted to encode several chaperone proteins, membrane transporters, lipases, and proteins involved in chitin metabolism with possible roles in cold tolerance. This genomic study updated the *C. rosea* IK726 genome, established a population genomic dataset for *C. rosea* and revealed important genomic regions for future studies of cold tolerance in this very promising BCA.

## Materials and methods

### Fungal growth and culture conditions

*Clonostachys rosea* strains (Supplementary Table 1) were revived from glycerol stocks stored at −80°C, and maintained on potato dextrose agar (PDA; Oxoid, Cambridge, UK) at 25°C in darkness. Two growth rate assays were performed in triplicates for each strain by inoculating ½ strength PDA petri dishes with an agar plug with actively growing mycelium, followed by incubation at 10°C and 25°C, respectively, in darkness. Growth measurements of strains at 10°C were made by marking the edges of the fungal mycelium in four places in a square shape on the petri dish at 4 days and 28 days post inoculation. The mean change in millimeters between the 4-day and 28-day marks was measured and divided by 24 to provide data as mm growth per day. Growth measurements at 25°C were done in the same manner, but measured from the edge of the inoculation agar plug to the mycelial front at 5 days post inoculation. Growth rate data at 10°C were analyzed by analysis of variance (ANOVA) using the Welch's test implemented in Minitab ver. 18 (Minitab Inc., State Collage, PA), while pairwise comparisons were made using the Games-Howell method at the 95% significance level. Growth rate data at 25°C were analyzed by ANOVA using a general linear model approach implemented in Minitab ver. 18 (Minitab Inc., State Collage, PA). Pairwise comparisons were made using the Fisher method at the 95% significance level. Linear regression analysis was performed by Pearson correlation in Minitab ver. 18 (Minitab Inc., State Collage, PA). Principle component analysis, using the R programming language, was performed in order to detect whether there was any correlation between geographical location of isolate origin and growth at 10°C.

For DNA extraction, strains were grown in 200 ml liquid Czapek-Dox medium (Sigma-Aldrich, Steinheim, Germany), Vogel's minimal medium (Vogel 1956) or malt extract (1.75 %) with peptone (0.25 %) medium at room temperature, shaking at 120 rpm. Cultures were harvested after 3–13 days, depending on growth rate, by snap freezing in liquid nitrogen, and then freeze-dried.

### Genomic DNA extraction and sequencing

High-quality genomic DNA was extracted using CTAB/chloroform extraction for Illumina sequencing or Qiagen-tip 100 (Qiagen, Hilden, Germany) for PacBio sequencing (Menkis et al., 2015). Starting material for both protocols were freeze-dried mycelium ground with sand in a mortar and pestle. The CTAB/chloroform was scaled up to 50 ml falcon tubes with 10 ml CTAB-buffer and two to four rounds of chloroform extraction. Qiagen-tip 100 DNA extractions followed the Qiagen genomic DNA handbook with modifications of the yeast protocol. Instead of making spheroplasts, extraction started with adding G2 buffer to the ground mycelium and thereafter following the protocol. Regardless of protocol, at the precipitation step DNA was harvested with a glass hook and the remaining DNA was pelleted by centrifugation. For PacBio sequencing the harvested DNA was used, while for Illumina sequencing the harvested DNA was mostly used but the precipitated DNA was used in a few cases. DNA quality and concentrations were measured with Nanodrop (Thermo Fisher Scientific, Waltham, MA), Qubit dsDNA BR assay kit (Thermo Fisher Scientific, Waltham, MA) and agarose gel electrophoresis.

Base coverage of the *C. rosea* IK726 genome was generated using PacBio RSII Technology with an insert length of 20 kbp using standard library preparation kits. Base coverage of additional *C. rosea* genomes were generated using Illumina HiSeqX paired end sequencing with an insert length of 350 bp and read length of 150 bp using standard library preparation kits. The PacBio long reads were assembled into polished contigs using HGAP and the SMRT workflow (Chin et al., 2013).

### Genome assembly and annotation, SNP calling and population structure analysis

The previously reported genome assembly of *C. rosea* IK726 (Karlsson et al., 2015) was combined with the polished contig output of the PacBio sequencing using quickmerge (Chakraborty et al., 2016). The PacBio contigs and the original assembly were compared using MUMmer ver. 3.23 (Delcher et al., 2003). The merged genome (version 2) was annotated using a pipeline described previously (Karlsson et al., 2015). For the additional 52 *C. rosea* strains, Illumina reads were aligned against the *C. rosea* IK726 ver. 2 genome using Bowtie 2 ver. 2.2.4 (Langmead and Salzberg, 2012). The alignments were further analyzed with SAMtools ver. 0.1.18 for filtering out PCR duplicates (Li et al., 2009). Variant calling was performed on filtered alignments using a combination of Freebayes ver. 1.0 and the bamaddrg script for adding read groups to binary alignment map (BAM) files (Garrison and Marth, 2012; Garrison, 2018). Population structure analysis was performed on the 53 isolates using Structure ver. 2.3.4 (Hubisz et al., 2009) using 8,000 randomly sampled SNPs across the population. Linkage disequilibrium (LD) was determined by using vcftools ver. 0.1.15, and to calculate *r*^2^ using a 10 kbp window (Danecek et al., 2011).

### Genome-wide association study

The GWAS was performed using PLINK ver. 1.90 (Purcell et al., 2007), using the parameters—maf 0.1—hwe 1e-5 for SNP filtering. The SNPs were annotated using the ANNOVAR software (Wang et al., 2010). The resulting beta and beta standard deviation from the PLINK analysis were used as input to the R package ashr, for empirical Bayesian multiple hypothesis testing, for estimating local false sign rate (lfsr) for more appropriate significance estimation (Stephens, 2016). We used an lfsr of 0.05 as cut-off for significance. In addition, we used false discovery rate (FDR) correction for multiple testing as implemented in PLINK. Identification of conserved protein modules and features were made using the conserved domain database (CDD, Marchler-Bauer et al., 2017) and the transporter classification database (TCDB, Saier et al., 2016).

### Chitin synthase phylogenetic analysis

The amino acid sequences of the *C. rosea* chitin synthetases CRV2G00008512 and CRV2G00019295 were used to search GenBank at the National Center for Biotechnology Information (NCBI) for similar sequences using the BlastP algorithm. Amino acid sequence alignment was performed using MUSCLE (Edgar, 2004; Tamura et al., 2013), in the MEGA6 software. Phylogenetic analysis was performed using Maximum Likelihood methods implemented in MEGA6. Pairwise gap deletion was performed and the substitution model used was WAG with gamma distributed rates. Statistical support was performed using 1,000 bootstrapped resamplings of the tree.

### Nucleotide accession numbers

Sequencing analysis files for all isolates in this study except *C. rosea* IK726 (deposited separately described by Karlsson et al., 2015) were deposited under the study accession number PRJEB26874 at the European Nucleotide Archive/European Molecular Biology Laboratory–European Bioinformatics Institute repository. PacBio sequencing files for the re-sequencing of *C. rosea* strain IK726, and the updated genome annotation were also deposited under ENA project PRJEB26874.

## Results

### Growth rates at 10°C and 25°C differs between *C. rosea* strains

Growth rates of 53 *C. rosea* strains at 10°C were normally distributed, ranged from 0.066 to 0.413 mm/day (Table 1), and there were significant differences between strains (*P* < 0.001). The lowest growth rates were recorded for strains CBS 708.97, SHW-1-1, 1881, and IK726 originating from USA, China, Slovenia and Denmark, respectively. The highest growth rates were recorded for two strains from Slovenia (1832 and 1882) and strain CBS 188.33 from the Netherlands. No connection between growth rate and geographic origin was detected (Supplementary Figure 1). Growth rates at 25°C were normally distributed, ranged from 0.940 to 3.796 mm/day (Table 1), and there were significant differences between strains (*P* < 0.001). There was a weak correlation between growth rates at 10°C and 25°C (*r*^2^ = 0.178, *P* = 0.025), indicating that the mechanisms for cold tolerance were distinct from the basis for high growth rate at ambient temperature. Therefore, the growth rate at 10°C for each strain was normalized by the growth rate at 25°C (Table 1) for the GWAS analysis, to enable identification of SNPs and genomic regions specifically contributing to higher growth rate at colder temperature.

**Table 1:** Growth rates of *Clonostachys rosea* strains on PDA at 10°C and 25°C, and adjusted growth at 10°C.

**Isolate**	**Cold growth mm/day**	**PDA growth mm/day**	**Cold rate/PDA rate**	**Origin**
GG-1-2	0.201	0.940	0.214	Asia
2177	0.326	1.556	0.210	Europe
CBS 705.97	0.399	2.202	0.181	North America
JLB-7-1	0.347	1.923	0.181	Asia
IK216	0.326	1.811	0.180	Europe
2178	0.358	2.020	0.177	Europe
1832	0.410	2.416	0.170	Europe
2176	0.295	1.755	0.168	Europe
1316	0.326	2.022	0.161	Europe
IK371	0.309	2.035	0.152	Europe
B13	0.396	2.683	0.148	Oceania
1701	0.260	1.881	0.138	Europe
CBS 188.33	0.410	3.000	0.137	Europe
CBS 376.55	0.399	2.952	0.135	North America
CBS 193.94	0.372	2.850	0.130	South America
GL-1-1	0.313	2.474	0.126	Asia
1882	0.413	3.290	0.126	Europe
1829	0.194	1.550	0.125	Europe
1833	0.233	1.857	0.125	Europe
CBS 178.28	0.333	2.800	0.119	Europe
CBS 287.78	0.226	1.921	0.117	North America
CMI192798	0.344	2.930	0.117	Europe
GJS89-34	0.295	2.525	0.117	South America
CBS 154.27	0.326	2.828	0.115	North America
CBS 907.72E	0.351	3.061	0.115	Asia
CBS 907.72D	0.281	2.458	0.114	Asia
1884	0.208	1.856	0.112	Europe
CBS 100502	0.233	2.141	0.109	Europe
CBS 148.72	0.278	2.588	0.107	Europe
1827	0.174	1.627	0.107	Europe
SDT-5-1	0.208	1.985	0.105	Asia
CBS 421.87	0.285	2.825	0.101	Europe
CBS 222.93	0.219	2.210	0.099	South America
1830	0.215	2.175	0.099	Europe
SYP-4-2	0.191	1.971	0.097	Asia
1883	0.278	2.892	0.096	Europe
CBS 704.97	0.160	1.724	0.093	North America
NHH-61-2	0.188	2.074	0.090	Asia
STG-21-1	0.142	1.586	0.090	Asia
CBS 569.69	0.191	2.200	0.087	Europe
JXLS-1-1	0.267	3.240	0.083	Asia
CBS 649.8	0.194	2.383	0.082	Africa
SHW-1-1	0.122	1.561	0.078	Asia
IBT7519	0.194	2.531	0.077	Europe
CBS 100000	0.201	2.776	0.073	Oceania
NHH-48-2	0.174	2.426	0.072	Asia
CBS 907.72G	0.222	3.315	0.067	Asia
1885	0.174	2.700	0.064	Europe
CBS 706.97	0.184	2.969	0.062	North America
CBS 277.5	0.188	3.045	0.062	North America
1881	0.128	2.446	0.053	Europe
IK726	0.132	3.138	0.042	Europe
CBS 708.97	0.066	2.002	0.033	North America

### PacBio sequencing results in an improved genome assembly of *C. rosea* IK726

PacBio sequencing of the *C. rosea* IK726 genome resulted in 71.2 Mbp sequence data. The PacBio reads were subjected to error correction of the longest reads by subread filtering, mapping and *de novo* assembly of all reads into long polished contigs using HGAP ver 3.0 and the SMRT pipeline (Chin et al., 2013; Roberts et al., 2013). Combining the polished contigs of the PacBio sequencing with the previously reported Illumina-based genome of *C. rosea* IK726 (Karlsson et al., 2015), expanded the genome from 58.3 (ver. 1) to 70.7 (ver. 2) Mbp across 767 scaffolds (Supplementary Table 2). N50 increased from 790 kbp to 1.9 Mbp, and the gap length decreased from 924 to 207 kbp (Supplementary Table 2). Comparisons between ver. 1 and ver. 2 using MUMmer revealed a strong correlation between the longer PacBio contigs and the ver. 1 genome (Supplementary Figure 2). Several smaller PacBio contigs were left without a corresponding scaffold of the old version, demonstrating the superior coverage generated by PacBio. A total of 21,246 genes were predicted, although this number dropped to 17,508 when excluding exclusively *ab initio* predicted genes.

### GWAS identifies SNPs associated with growth rate at 10°C

**Figure 1:**
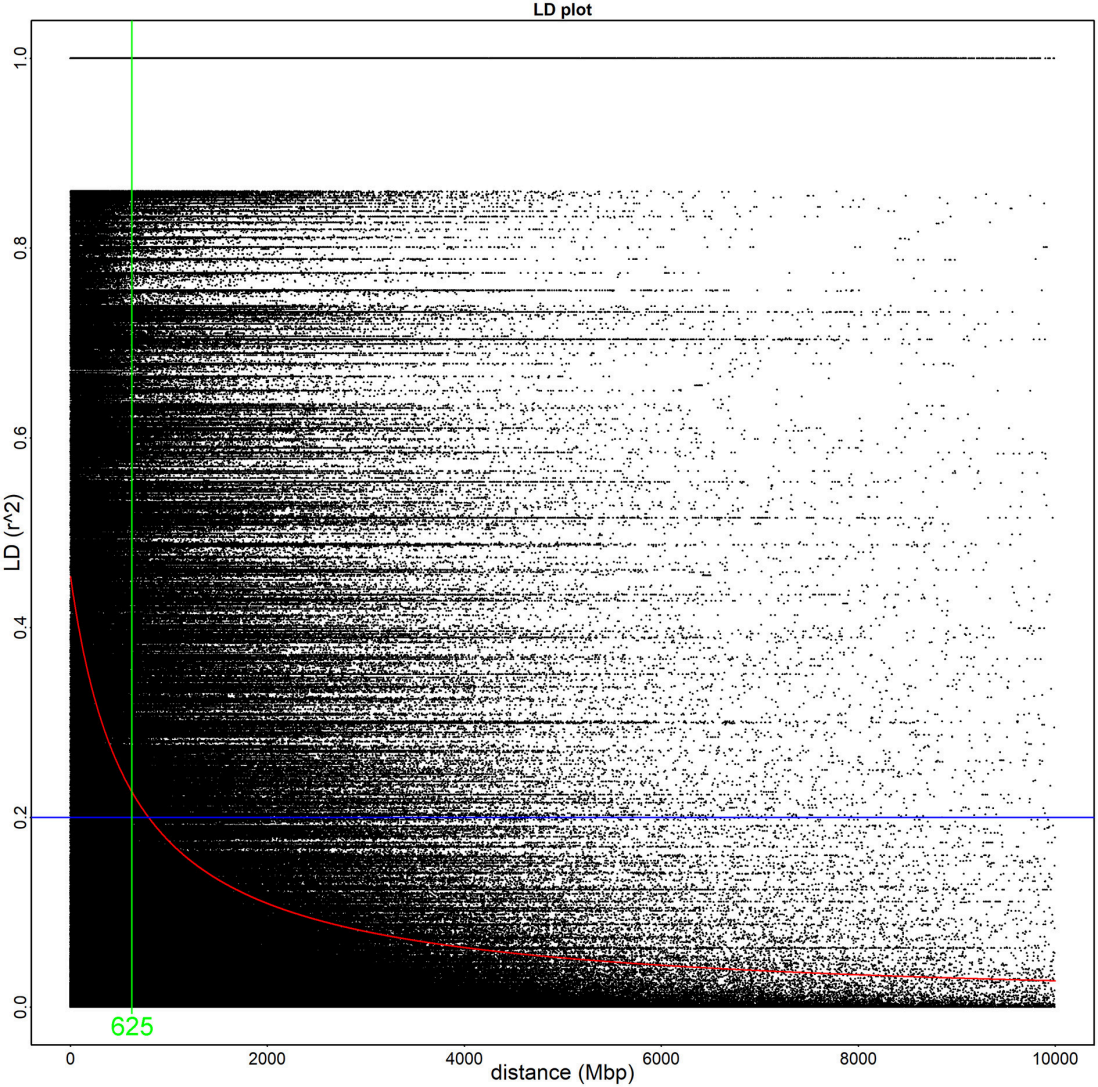
The linkage disequilibrium half decay is 625 base pairs in this population of *Clonostachys rosea*. Linkage disequilibrium of all identified SNPs in a 10,000 base pair window. The green line demonstrates the linkage disequilibrium half decay mark, the blue line highlights an *r*^2^ value of 0.2, and the red line describes the overall *r*^2^ decay according to the Hill and Weir formula.

**Figure 2:**
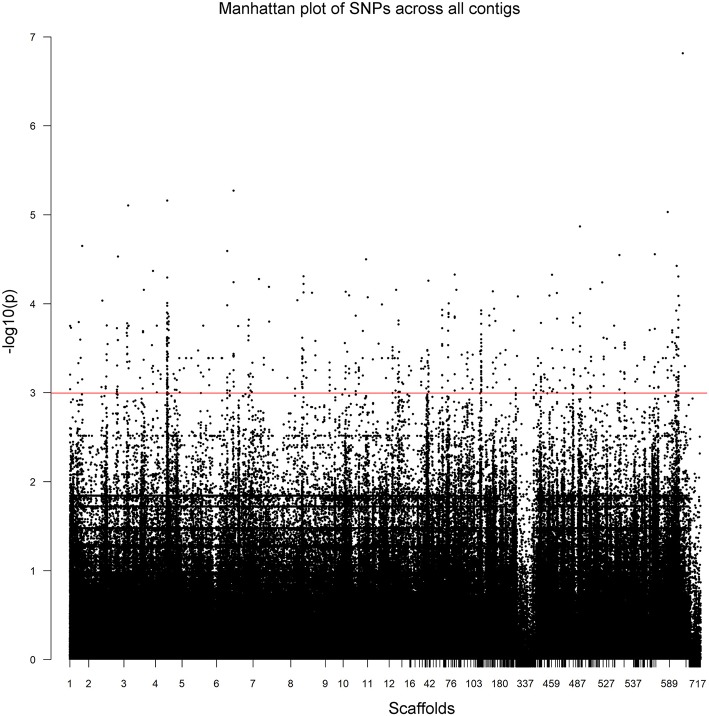
Unfiltered SNPs in *Clonostachys rosea* across all scaffolds, numbered as described in Supplementary Table 3. Manhattan plots of the scaffold positions and *P*-values of cold growth for all SNPs across the all scaffolds. The red line indicates a *P*-value of 0.05-log_10_.

The 52 Illumina-sequenced genomes exhibited an average alignment percentage to the *C. rosea* genome ver. 2 of 91% (Supplementary Table 1). The genomes had an average of 2.6 Gbp of total reads with a Q value of ≥30, resulting in an average coverage of 35X. Initially, we used vcftools to extract a total of 1,898,673 SNPs. These were further filtered during the GWAS analysis with PLINK, filtering SNPs using a minor allele frequency cut-off of 10% and a Hardy-Weinberg statistic of 10^−5^, leaving 63,726 SNPs. Based on 8,000 randomly sampled SNPs from the vcftool results, no population structure among the 53 strains was detected using the Structure software. The LD half decay distance was determined to 625 bp (Figure 1). Overall, the unfiltered SNPs were identified across 717 scaffolds (Figure 2, Supplementary Table 3).

Empirical Bayesian multiple hypothesis testing identified an association between 1,478 SNP markers and normalized growth rate at 10°C with an lfsr lower than 0.05 (Supplementary Table 4). These 1,478 SNPs were located on 227 different scaffolds (Supplementary Table 4). A single SNP (unitig_823:6016) was identified as associated with normalized growth rate at 10°C using FDR correction (Supplementary Table 4).

### Gene content in genomic regions associated with growth rate at 10°C

Out of the 1,478 significantly cold growth associated SNP markers, 581 were located within or close (< 1,000 bp) to 265 different genes, mostly predicted to encode proteins with unknown function (Supplementary Table 4). Out of these, 175 SNPs were located in exonic regions, 37 in intronic regions, 2 in splice sites, and 40 in the untranslated regions (UTRs) on the 3′ or 5′ ends. 145 of the 1,478 SNPs were highly significant according to the ashr analysis (lfsr < 10^−10^). The 20 most significant SNPs based on ashr are depicted in Table 2. The most common gene ontology categories were catalytic activity (GO:0003824), protein binding (GO:0005515), metabolic process (GO:0008152), oxidoreductase activity (GO:0016491), oxidation-reduction process (GO:0055114), ATP binding (GO:0005524), and zink ion binding (GO:0008270). The most highly associated SNP to growth rate at 10°C according to standard PLINK association (*P* = 1.5^*^10^−7^, FDR = 0.01) was located in gene CRV2G00015476. This gene was predicted to encode a beta lactam utilization protein, LamB. Nineteen SNPs were identified in an intergenic region between a gene for a predicted short-chain dehydrogenase (CRV2G00018099) and a gene for a predicted glutathione-dependent formaldehyde-activating enzyme (CRV2G00018100).

**Table 2:** Top 20 highest significance SNPs reported to be associated with growth rate at 10°C in ashr analysis.

**SNP**	**lfsr**	**Location**	**Location**	**Annotation**	**NCBI Conserved domain search**
unitig_630:6733	0.00	intergenic	CRV2G00019736(dist = 3623)	Protein of unknown function	No conserved domains identified
unitig_878:353	0.00	intergenic	NONE	.	.
unitig_760:385	0.00	intergenic	CRV2G00020684(dist = 6578)	Similar to FRQ Frequency clock protein	FRQ domain
unitig_47:563	0.00	intergenic	NONE	.	.
unitig_47:602	0.00	intergenic	NONE	.	.
unitig_793:2158	0.00	intergenic	CRV2G00016485(dist = 1475)	Protein of unknown function	No conserved domains identified
unitig_793:2167	0.00	intergenic	CRV2G00016485(dist = 1484)	Protein of unknown function	No conserved domains identified
unitig_298:2434	0.00	intergenic	NONE	.	.
unitig_298:2460	0.00	intergenic	NONE	.	.
unitig_280:2479	0.00	intergenic	NONE	.	.
unitig_345:10970	0.00	intergenic	CRV2G00021787(dist = 1241)	Protein of unknown function	FN3-like domain
unitig_246:11272	0.00	exonic	CRV2G00021642	Similar to fmpE Nonribosomal peptide synthetase	AFD class I superfamily, alpha am amid superfamily
unitig_640:12334	0.00	exonic	CRV2G00022020	Protein of unknown function	Fungal_TF_MHR, GAL4, DNA pol3 gamma3 superfamily
unitig_640:12340	0.00	exonic	CRV2G00022020	Protein of unknown function	Fungal_TF_MHR, GAL4, DNA pol3 gamma3 superfamily
unitig_818:85374	0.00	intergenic	CRV2G00017605(dist = 1606)	Mitochondrial chaperone BCS1-B	UhpC superfamily
unitig_601:93320	0.00	intergenic	CRV2G00019351(dist = 7767)	Protein of unknown function	Fasciclin domain
unitig_643:109199	0.00	intergenic	CRV2G00019327(dist = 50251)	Similar to betA Oxygen-dependent choline dehydrogenase	Choline dehydrogenase
unitig_697:153157	0.00	upstream	CRV2G00018410(dist = 872)	Protein of unknown function	No conserved domains identified
unitig_823:628705	0.00	intergenic	CRV2G00015724(dist = 3408)	Similar to FUS6 Efflux pump FUS6	TRI12 Superfamily
scf_014:970797	0.00	intergenic	CRV2G00012293(dist = 2794)	Protein of unknown function	GH18 chitinase-like superfamily

Ten and eight SNPs, respectively, were identified close, exonic or within 1,000 bp up- or downstream, to two genes predicted to encode mitochondrial chaperone proteins (CRV2G00019610 and CRV2G00008672) (Supplementary Table 4). Two additional genes encoding heat shock protein (HSP) chaperones contained exonic SNPs; the small ATP-independent HSP chaperone CRV2G00019134 and the ATP-dependent HSP70 chaperone CRV2G00021296. Four SNPs were identified close, exonic, or within 500 bp upstream, to a gene for a predicted efflux membrane protein (CRV2G00021344). A more detailed analysis revealed that is was a major facilitator superfamily (MFS) transporter from the drug:H^+^ antiporter-2 family (2.A.1.3), with the highest sequence similarity to members from the 2.A.1.3.33, 2.A.1.3.65, and 2.A.1.3.73 subfamilies that are implicated in drug, fungicide, or fungal secondary metabolite export. We also identified SNPs nearby (< 700 bp) two additional genes putatively encoding MFS efflux pumps from the same drug:H+ antiporter-2 family (CRV2G00015724 and CRV2G00018834), also implicated in drug resistance. Other transport protein-encoding genes associated with significant SNPs were CRV2G00019382 and CRV2G00018067 encoding homologs of *Saccharomyces cerevisiae* FMP42, possibly associated with mitochondria.

**Figure 3:**
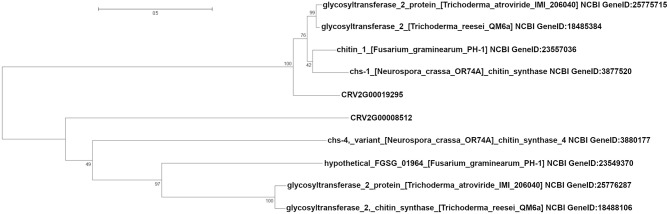
Phylogenetic analysis of *Clonostachys rosea* chitin synthases. Amino acid sequences of selected chitin synthases were aligned using MUSCLE and a phylogenetic analysis conducted using maximum likelihood methods. Bootstrapped branch statistics values are depicted above the branches. The bar indicates mean number of amino acid substitutions per site. Protein identifiers include NCBI Gene ID number.

We also identified two different genes encoding putative esterases/lipases (CRV2G00019392, CRV2G00021710) with three and one SNPs, respectively, exonic or within 600 bp downstream, significantly associated with growth rate at cold temperature. We detected seven SNPs close to or within three genes associated with chitin metabolism; CRV2G00008512, CRV2G00019295 (chitin synthases), and CRV2G00019292 (the *chiA4* chitinase). Additionally, we identified CRV2G00012293 (chitinase domain containing protein) with a significantly cold growth associated SNP at a distance of 2794 bp (Table 2). A sequence analysis of CRV2G00008512 and CRV2G00019295 showed that they displayed similarity with chitin synthases from other *Sordariomycete* fungi (Supplementary Table 5). A phylogenetic analysis further revealed that CRV2G00019295 clustered together with chitin synthase 1 from *N. crassa*, while CRV2G00008512 was more distantly related with chitin synthases from other Sordariomycetes (Figure 3). Besides the two previously mentioned mitochondrial chaperone protein genes and the FMP42 transporters, additional genes in close proximity with significant SNPs were predicted to encode proteins related to mitochondrial functioning; CRV2G00021544 putatively encoding a mitochondrial kinase activator of the bc1 complex required for the biosynthesis of Coenzyme Q and CRV2G00019380 similar to a mitochondrial aminolevulinate synthase involved in heme biosynthesis (Supplementary Table 5). We also detected three SNPs associated (one 6578 bp upstream, two in the 5′ UTR region) with CRV2G00020684, and one SNP associated with CRV2T00007803 (exonic), associated with frequency clock protein (FRQ) encoding gene in *C. rosea* (Supplementary Table 4, Table 2).

## Discussion

Fungi exhibit several advantages for applying GWA analysis such as small genomes, a haploid life stage and production of high numbers of clonal propagules that can be used for repeated phenotypic assessments. Especially the possibility to genotype haploid individuals by sequencing makes GWAS particularly powerful, since the phenotype is the results of a single haplotype and not a result of two haplotypes as in the diploid case where heterozygosity can confound the signal. Despite these advantages, GWA analyses have not yet been utilized to study the genetic basis for biocontrol traits in fungal BCAs. In this study, we therefore set out to establish *C. rosea* as a population genomic model system for BCA fungi and to evaluate its usefulness for GWAS.

A high-quality reference genome with few gaps is an important aspect for a successful GWAS. The PacBio sequencing platform has been shown to improve older assemblies, primarily for eukaryotic organisms, by reconciling the long reads with older second generation short read sequencers (Chakraborty et al., 2016; Jayakumar and Sakakibara, 2017). Combining PacBio polished contigs with Illumina-based sequences substantially increased N50 and decreased gaps in the *C. rosea* ver. 2 genome. It also increased the total genome size to 70.7 Mbp, which is considerably larger than the genomes of other hypocrealean mycoparasitic species (Karlsson et al., 2018) such as *Trichoderma* spp. (31.7–39.0 Mbp, Kubicek et al., 2011; Studholme et al., 2013; Xie et al., 2014), *Tolypocladium ophioglossoides* (31.2 Mbp, Quandt et al., 2015) and *Escovopsis weberi* (29.5 Mbp, de Man et al., 2016). The draft genome of the closely related species *C. chloroleuca* (Moreira et al., 2016) is 55.4 Mbp (Sun et al., 2015), although this was based on Illumina-technology only and therefore comparable to the *C. rosea* ver. 1 genome of 58.3 Mbp (Karlsson et al., 2015). The large genomes of *Clonostachys* spp. is however comparable to certain related *Fusarium* spp., such as *F. solani* (51.1 Mbp, Coleman et al., 2009) and *F. oxysporum* (61.4 Mbp, Ma et al., 2010). The *C. rosea* ver. 2 genome was predicted to contain 21,246 genes that were 6,978 more than ver. 1 (Karlsson et al., 2015). However, 3,738 of these genes were solely predicted through *ab initio* methods and some of these may represent artifacts, suggesting that the total gene number in *C. rosea* is between 17,508 and 21,246.

We further established a population genomic dataset by sequencing the genomes of 52 different *C. rosea* strains. The high coverage of the Illumina-based genomes and the high sequence similarity with the strain IK726 ver. 2 genome indicate high quality of the generated genomes. Previous fungal GWASs have used both smaller and larger populations to successfully associate SNP markers with virulence in *H. annosum* (23 strains), *Z. tritici* (106 strains), *P. nodorum* (191 strains), with virulence, deoxynivaleol production and azole sensitivity in *F. graminearum* (119 strains) and with azole sensitivity in *Rhynchosporium commune* (120 strains) (Dalman et al., 2013; Gao et al., 2016; Mohd-Assaad et al., 2016; Talas et al., 2016; Hartmann et al., 2017).

Another requirement of successful GWA analyses is the presence of enough recombination events in the population. The homothallic reproductive mode of *C. rosea* (Schroers et al., 1999) may be an obstacle for this type of analysis if repeated selfings between identical genomes results in an essentially clonal population. However, the high genetic variation and rapid LD decay detected in our worldwide *C. rosea* population is a strong indication of reoccurring outcrossing between genetically different strains, showing that *C. rosea* is a facultative homothallic species. Genetically distinct *C. rosea* individuals have been isolated from the same field in Denmark (Bulat et al., 2000), indicating local genetic variation. Another homothallic species, *Aspergillus nidulans*, has been shown to occasionally outcross, resulting in genetic recombination within populations (Kronstad, 2007; López-Villavicencio et al., 2013). Recombination and possible outcrossing has also been reported for *Aspergillus fumigatus* and *Letharia* species (Kroken and Taylor, 2001; Pringle et al., 2005).

High variation in growth rate at 10°C in *C. rosea*, with no connection with the geographic origin of the strains and the lack of genetic population structure indicate that a GWAS of the trait is possible. High growth rate at lower temperature may be an important trait for BCAs, especially for rapid colonization of new substrates with limited amounts of competing microorganisms such as wounds, flowers, and developing roots (Jensen et al., 2016). The connection between cold tolerance and interference competition through mycoparasitism or antibiosis (Jensen et al., 2017) is less well established. In fact, *C. rosea* strain IK726 was among the most slow-growing strains at 10°C in our study, at the same time as it repeatedly has been shown to be a very efficient BCA against several different fungal plant pathogenic species (Jensen et al., 2007).

The normal distribution of the cold tolerance trait in *C. rosea* further suggests a polygenic inheritance that depends on several different genes. Many medically and agriculturally relevant traits are in fact multigenic and it has been notoriously difficult to achieve a reliable estimation of the true effect size of individual loci to genetic variation contributing to polygenic phenotypic differences (Boyle et al., 2017). Thus, the emerging view of the genome as a uniform distribution of contributing, however small, sequence variants to different phenotypes, the “omnigenic” model, highlights the propensity to underestimate the true significance of SNPs and similar genomic variations on a trait (Boyle et al., 2017). So, in line with the omnigenic trait view of the genome, we used the R package ashr that produces SNPs with a true effect size of zero or non-zero (Stephens, 2016). Recently, ashr has been used in some GWASs as an empirical Bayes large scale hypothesis testing alternative to false discovery rate analysis, by using effect size and its standard error, instead of using only one parameter, such as *P*-values (Stephens, 2016; Boyle et al., 2017; Petit et al., 2017). The approach by Stephens (2016) has been proposed to be more robust when dealing with large scale hypothesis testing, producing a local false sign rate (lfsr) as an analogous alternative to false discovery rate. The use of ashr allowed us to identify 1,478 SNPs significantly associated with *C. rosea* growth rate at 10°C, located physically (< 1000 bp) close to about 265 genes.

As *C. rosea* is found globally, an evolved ability for cold tolerance would be integral for a generalist lifestyle across climates (Knudsen et al., 1995; Robinson, 2001; Abreu et al., 2014; Karlsson et al., 2015; Moreira et al., 2016). One important reason for wanting to study cold tolerance refers to the survival in temperate and arctic regions, as well as, in cold storage spaces, imperative for the long-term practical function and fitness of BCAs. The mechanisms for adapting to cold are numerous, and may include secretion of proteins with high catalytic efficiency active at low temperatures, transporters, lipid metabolism, secretion of secondary metabolites that counteract freezing damage of the membrane and intracellularly (Robinson, 2001; Blanc et al., 2012). Temperature and day length, depending on the geographical location, have been suggested to be major environmental factors impacting local adaptation of diverging *N. crassa* populations (Ellison et al., 2011). Significant SNPs were identified in or close to three genes encoding putative chaperone proteins. Regulation of mitochondrial chaperone proteins has been identified in association with temperature adaptation in diverse organisms such as the moth *Epiblema scudderiana* and the fungus *S. cerevisiae* (Schmitt, 1996; Lyons et al., 2015; Zhang et al., 2018). As chaperone proteins have also been discussed as important for cold tolerance in plants as well, this result demonstrates an interesting basis for further studies of chaperone importance in cold stress in *C. rosea* (Al-Whaibi, 2011). Several other genes putatively encoding proteins related to the mitochondria were identified by the GWAS, and further emphasize the importance of mitochondrial functioning for abiotic stress tolerance. The importance of mitochondrial activity in response to cold stress has been discussed in both animals and plants (Mollica et al., 2005; Fangue et al., 2009; Karami-Moalem et al., 2018).

Chitin is an important component of the fungal cell wall, and balancing chitin synthesis and degradation provides the appropriate plasticity and rigidity needed for hyphal growth (Specht et al., 1996). Therefore, it was interesting to find two putative chitin synthase genes and the *chiA4* chitinase gene among the genes associated with cold tolerance. The *chiA4* gene was reported to be constitutively expressed in *C. rosea* (Tzelepis et al., 2015), which fits well with a role in cell wall modeling during growth. Chitinases have also been implicated in cold tolerance in plants (Nakamura et al., 2008; Kashyap and Deswal, 2017). Certain fungal chitin synthases have also been associated with adaptation to temperature stress (Lenardon et al., 2009; Liu et al., 2017). The chitin synthase encoded by CRV2G00019295 was found to be most related to chs-1 in *N. crassa*, which has been found to play a major role in cell wall biogenesis (Yarden and Yanofsky, 1991).

Three different putative membrane efflux transporter genes from the MFS drug:H+ antiporter-2 family also contained SNPs associated with cold tolerance. Both the MFS transporter families 2.A.1.3.33 and 2.A.1.3.65 are shown to evolve under selection for increased gene copy numbers in *C. rosea*, emphasizing the importance for membrane transport in the lifestyle of *C. rosea* (Nygren et al., 2018). The connection between membrane transport and cold tolerance is less established. Efflux pumps have been associated with cold stress adaptation in bacteria, specifically by *Moraxella catarrhalis*, where cold stress reportedly activates transcription and efflux activity the *AcrAB-OprM* system (Spaniol et al., 2015). Furthermore, studies indicate a connection between ions, secondary metabolite production (such as flavonoids) and membrane transport with cold tolerance in animals and plants (Kaplan, 2004; Wei et al., 2006; Storey and Storey, 2012; Wang M. et al., 2013; Wang X. C. et al., 2013). Additionally, secondary metabolites such as melanin have proven to be important in adjustment to temperature for the fungus *R. commune* and *Cryptococcus neoformans* (Rosas and Casadevall, 1997; Zhu et al., 2018). Thus, differences in transporter effectiveness due to differing alleles between strains may impact the ability of fungi to adjust their osmotic equilibrium, concentration of important metabolites for protein stability and membrane stability.

Finally, indications of lipid modifications in *C. rosea* as a response to colder temperatures comes from the association of SNPs situated in several putative lipases. Lipid structure and concentration is important for cold adaptation in many organisms (Arthur and Watson, 1976; Upchurch, 2008; van Dooremalen et al., 2011; Treseder and Lennon, 2015) and further studies of the association between the lipases and cold tolerance *C. rosea* is warranted. Comparative genomics and a growth assay at 10°C of *N. crassa* has suggested that the FRQ protein may be of significance in cold resistance, in part due to the link between lower temperatures and less daylight hours across geographical location (Ellison et al., 2011). From the GWAS we detected four significant SNPs associated with two genes in *C. rosea* encoding predicted FRQ proteins with some homology (~80–90% cover, 33–36% identities) to *N. crassa* FRQ; CRV2G00020684 (one SNP 6578 bp upstream, two in the UTR5 region) and CRV2G00007803 (one exonic SNP). These results support the link between growth in cold and FRQ (Table 2, Supplementary Table 4).

We here present an improved genome assembly of *C. rosea* strain IK726 and whole-genome re-sequencing of an additional 52 *C. rosea* strains, thereby establishing *C. rosea* as the premier model species for genomic investigations of fungal BCAs. We further show that GWAS is a powerful tool for studying the genetic basis for phenotypes related with biocontrol activity. We identified an association between cold tolerance and SNPs residing in multiple genes, encoding putative mitochondrial chaperone and abiotic stress proteins, membrane efflux transport proteins, and lipases. These genes are of interest for further studies of cold stress and potential biocontrol efficiency in temperate climates.

## Author contributions

All authors were involved in planning the study and writing the manuscript. M-HS, S-DL, and H-JS performed identification of the strains, KI and MD performed fungal growth and maintenance, and KI performed DNA extraction and preparation. MB and MK performed the growth assays. MB, MBD, and MK performed the genome sequencing analysis and GWAS analysis. DJ, MBD, and MK secured the funding.

### Conflict of interest statement

The authors declare that the research was conducted in the absence of any commercial or financial relationships that could be construed as a potential conflict of interest.

## Supplementary material

Supplementary Figure 1 Origin of strains does not correlate with growth on PDA at 10°C. Using principal component analysis, we compared growth rates on PDA at 10°C, PDA at room temperature, and adjusted growth at 10°C to room temperature. The analysis revealed no correlation to the strains' origin of isolation.

Supplementary Figure 2 MUMmer alignment output of PacBio long contigs to *Clonostachys rosea* IK726 ver. 1 genome scaffolds.

Supplementary Table 1 List of all *Clonostachys rosea* isolates sequenced using the Illumina platform in this study, their origin, and Bowtie2 alignment rate.

Supplementary Table 2 Details of the assembled *Clonostachys rosea* IK726 genome version 2.

Supplementary Table 3 List of *Clonostachys rosea* genome version 2 scaffolds and their corresponding scaffold numbers in Figure 2.

Supplementary Table 4 Significant GWAS analysis results based on PLINK and ashr.

Supplementary Table 5 Protein sequences of chitin synthases used in the phylogenetic analysis in Figure 3.

## References

[B1] Abreu L. M. Moreira G. M. Ferreira D. Rodrigues-Filho E. Pfenning L. H. (2014). Diversity of Clonostachys species assessed by molecular phylogenetics and MALDI-TOF mass spectrometry. Fungal Biol. 118, 1004–1012. 10.1016/j.funbio.2014.10.001 25457948

[B2] Al-Whaibi M. H. (2011). Plant heat-shock proteins: a mini review. J. King Saud Univ. Sci. 23, 139–150. 10.1016/j.jksus.2010.06.022

[B3] Arthur H. Watson K. (1976). Thermal adaptation in yeast: growth temperatures, membrane lipid, and cytochrome composition of psychrophilic, mesophilic, and thermophilic yeasts. J. Bacteriol. 128, 56–68. 988016 10.1128/jb.128.1.56-68.1976 PMC232826

[B4] Blanc G. Agarkova I. Grimwood J. Kuo A. Brueggeman A. Dunigan D. D.. (2012). The genome of the polar eukaryotic microalga Coccomyxa subellipsoidea reveals traits of cold adaptation. Genome Biol. 13:R39. 10.1186/gb-2012-13-5-r39 22630137 PMC3446292

[B5] Boyle E. A. Li Y. I. Pritchard J. K. (2017). An expanded view of complex traits: from polygenic to omnigenic. Cell 169, 1177–1186. 10.1016/j.cell.2017.05.038 28622505 PMC5536862

[B6] Bulat S. A. Lübeck M. Alekhina I. A. Jensen D. F. Knudsen I. M. B. Lübeck P. S. (2000). Identification of a universally primed-PCR-derived sequence-characterized amplified region marker for an antagonistic strain of clonostachys rosea and development of a strain-specific PCR detection assay. Appl. Environ. Microbiol. 66, 4758–4763. 10.1128/AEM.66.11.4758-4763.2000 11055920 PMC92376

[B7] Chakraborty M. Baldwin-Brown J. G. Long A. D. Emerson J. J. (2016). Contiguous and accurate *de novo* assembly of metazoan genomes with modest long read coverage. Nucleic Acids Res. 44:e147. 10.1093/nar/gkw654 27458204 PMC5100563

[B8] Chin C. S. Alexander D. H. Marks P. Klammer A. A. Drake J. Heiner C.. (2013). Nonhybrid, finished microbial genome assemblies from long-read SMRT sequencing data. Nat. Methods 10, 563–569. 10.1038/nmeth.2474 23644548

[B9] Coleman J. J. Rounsley S. D. Rodriguez-Carres M. Kuo A. Wasmann C. C. Grimwood J.. (2009). The genome of nectria haematococca: contribution of supernumerary chromosomes to gene expansion. PLoS Genet. 5:e1000618. 10.1371/journal.pgen.1000618 19714214 PMC2725324

[B10] Dalman K. Himmelstrand K. Olson Å. Lind M. Brandström-Durling M. Stenlid J. (2013). A genome-wide association study identifies genomic regions for virulence in the non-model organism heterobasidion annosum s.s. PLoS ONE 8:e53525. 10.1371/journal.pone.0053525 23341945 PMC3547014

[B11] D'Amico S. Claverie P. Collins T. Georlette D. Gratia E. Hoyoux A.. (2002). Molecular basis of cold adaptation. Philos. Trans. R. Soc. B Biol. Sci. 357, 917–925. 10.1098/rstb.2002.1105 12171655 PMC1692995

[B12] Danecek P. Auton A. Abecasis G. Albers C. A. Banks E. DePristo M. A.. (2011). The variant call format and VCFtools. Bioinformatics 27, 2156–2158. 10.1093/bioinformatics/btr330 21653522 PMC3137218

[B13] de Man T. J. Stajich J. E. Kubicek C. P. Teiling C. Chenthamara K. Atanasova L.. (2016). Small genome of the fungus *Escovopsis weberi*, a specialized disease agent of ant agriculture. Proc. Natl. Acad. Sci. U.S.A. 113, 3567–3572. 10.1073/pnas.1518501113 26976598 PMC4822581

[B14] Delcher A. L. Salzberg S. L. Phillippy A. M. (2003). Using MUMmer to identify similar regions in large sequence sets. Curr. Protoc. Bioinform. 10:10.3. 10.1002/0471250953.bi1003s00 18428693

[B15] Edgar R. C. (2004). MUSCLE: multiple sequence alignment with high accuracy and high throughput. Nucleic Acids Res. 32, 1792–1797. 10.1093/nar/gkh340 15034147 PMC390337

[B16] Ellison C. E. Hall C. Kowbel D. Welch J. Brem R. B. Glass N. L.. (2011). Population genomics and local adaptation in wild isolates of a model microbial eukaryote. Proc. Natl. Acad. Sci. U.S.A. 108, 2831–2836. 10.1073/pnas.1014971108 21282627 PMC3041088

[B17] Fangue N. A. Richards J. G. Schulte P. M. (2009). Do mitochondrial properties explain intraspecific variation in thermal tolerance? J. Exp. Biol. 212, 514–522. 10.1242/jeb.024034 19181899

[B18] Gao Y. Liu Z. Faris J. D. Richards J. Brueggeman R. S. Li X.. (2016). Validation of genome-wide association studies as a tool to identify virulence factors in *Parastagonospora nodorum*. Phytopathology 106, 1177–1185. 10.1094/PHYTO-02-16-0113-FI 27442533

[B19] Garrison E. (2018). Bamaddrg: Adds Sample Names and Read-Group (RG) Tags to BAM Alignments. Available online at: https://github.com/ekg/bamaddrg (Accessed February 7, 2018).

[B20] Garrison E. Marth G. (2012). Haplotype-Based Variant Detection From Short-Read Sequencing. ArXiv12073907 Q-Bio. Available online at: http://arxiv.org/abs/1207.3907 (Accessed February 7, 2018).

[B21] Hartmann F. E. Sánchez-Vallet A. McDonald B. A. Croll D. (2017). A fungal wheat pathogen evolved host specialization by extensive chromosomal rearrangements. ISME J. 11, 1189–1204. 10.1038/ismej.2016.196 28117833 PMC5437930

[B22] Hubisz M. J. Falush D. Stephens M. Pritchard J. K. (2009). Inferring weak population structure with the assistance of sample group information. Mol. Ecol. Resour. 9, 1322–1332. 10.1111/j.1755-0998.2009.02591.x 21564903 PMC3518025

[B23] Jayakumar V. Sakakibara Y. (2017). Comprehensive evaluation of non-hybrid genome assembly tools for third-generation PacBio long-read sequence data. Brief. Bioinformatics. 10.1093/bib/bbx147 29112696 PMC6585154

[B24] Jensen D. F. Karlsson M. Lindahl B. D. (2017). Chapter 38: Fungal–fungal interactions: from natural ecosystems to managed plant production, with emphasis on biological control of plant diseases, in Mycology, eds Dighton J. White J. F. (Boca Raton, FL: CRC Press), 549–562. 10.1201/9781315119496-39

[B25] Jensen D. F. Karlsson M. Sarrocco S. Vannacci G. (2016). Biological control using microorganisms as an alternative to disease resistance, in Plant Pathogen Resistance Biotechnology, ed D. B. Collinge (Hoboken, NJ: John Wiley & Sons, Inc), 341–363. 10.1002/9781118867716.ch18

[B26] Jensen D. F. Knudsen I. M. B. Beck M. L. Mamarabadi M. Hockenhull J. Jensen B. (2007). Development of a biocontrol agent for plant disease control with special emphasis on the near commercial fungal antagonist *Clonostachys rosea* strain ‘IK726.' Australas. Plant Pathol. 36, 95–101. 10.1071/AP07009

[B27] Kaplan F. (2004). Exploring the temperature-stress metabolome of arabidopsis. Plant Physiol. 136, 4159–4168. 10.1104/pp.104.052142 15557093 PMC535846

[B28] Karami-Moalem S. Maali-Amiri R. Kazemi-Shahandashti S. S. (2018). Effect of cold stress on oxidative damage and mitochondrial respiratory properties in chickpea. Plant Physiol. Biochem. 122, 31–39. 10.1016/j.plaphy.2017.11.011 29172103

[B29] Karlsson M. Atanasova L. Jensen D. F. Zeilinger S. (2018). Necrotrophic mycoparasites and their genomes, in The Fungal Kingdom, eds Heitman, Howlett, Crous, Stukenbrock, James, and Gow (Washington, DC: ASM Press), 1005–1026.

[B30] Karlsson M. Durling M. B. Choi J. Kosawang C. Lackner G. Tzelepis G. D.. (2015). Insights on the evolution of mycoparasitism from the genome of Clonostachys rosea. Genome Biol. Evol. 7, 465–480. 10.1093/gbe/evu292 25575496 PMC4350171

[B31] Kashyap P. Deswal R. (2017). A novel class I chitinase from *Hippophae rhamnoides*: indications for participating in ICE-CBF cold stress signaling pathway. Plant Sci. 259, 62–70. 10.1016/j.plantsci.2017.03.004 28483054

[B32] Knudsen I. M. B. Hockenhull J. Jensen D. F. (1995). Biocontrol of seedling diseases of barley and wheat caused by *Fusarium culmorum* and *Bipolaris sorokiniana*: effects of selected fungal antagonists on growth and yield components. Plant Pathol. 44, 467–477. 10.1111/j.1365-3059.1995.tb01669.x

[B33] Kroken S. Taylor J. W. (2001). Outcrossing and recombination in the lichenized fungus letharia. Fungal Genet. Biol. 34, 83–92. 10.1006/fgbi.2001.1291 11686674

[B34] Kronstad J. W. (2007). Self-fertility: the genetics of sex in lonely fungi. Curr. Biol. 17, R843–R845. 10.1016/j.cub.2007.08.002 17925212

[B35] Kubicek C. P. Herrera-Estrella A. Seidl-Seiboth V. Martinez D. A. Druzhinina I. S. Thon M.. (2011). Comparative genome sequence analysis underscores mycoparasitism as the ancestral life style of Trichoderma. Genome Biol. 12:R40. 10.1186/gb-2011-12-4-r40 21501500 PMC3218866

[B36] Langmead B. Salzberg S. L. (2012). Fast gapped-read alignment with Bowtie 2. Nat. Methods 9, 357–359. 10.1038/nmeth.1923 22388286 PMC3322381

[B37] Lenardon M. D. Lesiak I. Munro C. A. Gow N. A. (2009). Dissection of the *Candida albicans* class I chitin synthase promoters. Mol. Genet. Genomics 281, 459–471. 10.1007/s00438-009-0423-0 19153767 PMC3468743

[B38] Li G. Q. Huang H. C. Kokko E. G. Acharya S. N. (2002). Ultrastructural study of mycoparasitism of Gliocladium roseum on *Botrytis cinerea*. Bot. Bull. Acad. Sin. 43, 211–218. 10.7016/BBAS.200207.0211

[B39] Li H. Handsaker B. Wysoker A. Fennell T. Ruan J. Homer N.. (2009). The sequence alignment/map format and SAMtools. Bioinformatics 25, 2078–2079. 10.1093/bioinformatics/btp352 19505943 PMC2723002

[B40] Li H.-Y. Shen M. Zhou Z.-P. Li T. Wei Y. Lin L. (2012). Diversity and cold adaptation of endophytic fungi from five dominant plant species collected from the baima snow mountain, southwest China. Fungal Divers. 54, 79–86. 10.1007/s13225-012-0153-1

[B41] Liu R. Xu C. Zhang Q. Wang S. Fang W. (2017). Evolution of the chitin synthase gene family correlates with fungal morphogenesis and adaption to ecological niches. Sci. Rep. 7:44527. 10.1038/srep44527 28300148 PMC5353729

[B42] López-Villavicencio M. Debets A. J. M. Slakhorst M. Giraud T. Schoustra S. E. (2013). Deleterious effects of recombination and possible nonrecombinatorial advantages of sex in a fungal model. J. Evol. Biol. 26, 1968–1978. 10.1111/jeb.12196 23848947

[B43] Lyons P. J. Crapoulet N. Storey K. B. Morin P. J. (2015). Identification and profiling of miRNAs in the freeze-avoiding gall moth Epiblema scudderiana via next-generation sequencing. Mol. Cell. Biochem. 410, 155–163. 10.1007/s11010-015-2547-3 26328872

[B44] Ma L. J. van der Does H. C. Borkovich K. A. Coleman J. J. Daboussi M. J. Di Pietro A.. (2010). Comparative genomics reveals mobile pathogenicity chromosomes in Fusarium. Nature 464, 367–373. 10.1038/nature08850 20237561 PMC3048781

[B45] Marchler-Bauer A. Bo Y. Han L. He J. Lanczycki C. J. Lu S.. (2017). CDD/SPARCLE: functional classification of proteins via subfamily domain architectures. Nucleic Acids Res. 45, D200–D203. 10.1093/nar/gkw1129 27899674 PMC5210587

[B46] Menkis A. Marčiulynas A. Gedminas A. Lynikien,e J. Povilaitien,e A. (2015). High-throughput sequencing reveals drastic changes in fungal communities in the phyllosphere of norway spruce (*Picea abies*) following invasion of the spruce bud scale (*Physokermes piceae*). Microb. Ecol. 70, 904–911. 10.1007/s00248-015-0638-z 26054703

[B47] Mohd-Assaad N. McDonald B. A. Croll D. (2016). Multilocus resistance evolution to azole fungicides in fungal plant pathogen populations. Mol. Ecol. 25, 6124–6142. 10.1111/mec.13916 27859799

[B48] Mollica M. P. Lionetti L. Crescenzo R. Tasso R. Barletta A. Liverini G.. (2005). Cold exposure differently influences mitochondrial energy efficiency in rat liver and skeletal muscle. FEBS Lett. 579, 1978–1982. 10.1016/j.febslet.2005.02.044 15792806

[B49] Moreira G. M. Abreu L. M. Carvalho V. G. Schroers H.-J. Pfenning L. H. (2016). Multilocus phylogeny of *Clonostachys* subgenus *Bionectria* from Brazil and description of *Clonostachys chloroleuca* sp. nov. Mycol. Prog. 15, 1031–1039. 10.1007/s11557-016-1224-6

[B50] Nakamura T. Ishikawa M. Nakatani H. Oda A. (2008). Characterization of cold-responsive extracellular chitinase in bromegrass cell cultures and its relationship to antifreeze activity. Plant Physiol. 147, 391–401. 10.1104/pp.106.081497 18359848 PMC2330313

[B51] Nygren K. Dubey M. Zapparata A. Iqbal M. Tzelepis G. D. Durling M. B.. (2018). The mycoparasitic fungus *Clonostachys rosea* responds with both common and specific gene expression during interspecific interactions with fungal prey. Evol. Appl. 11, 931–949. 10.1111/eva.12609 29928301 PMC5999205

[B52] Petit M. Astruc J. M. Sarry J. Drouilhet L. Fabre S. Moreno C.. (2017). Variation in recombination rate and its genetic determinism in sheep populations. Genetics 207, 767–784. 10.1534/genetics.117.300123 28978774 PMC5629338

[B53] Pringle A. Baker D. M. Platt J. L. Wares J. P. Latgé J. P. Taylor J. W. (2005). Cryptic speciation in the cosmopolitan and clonal human pathogenic fungus *Asperigillus fumigatus*. Evolution 59, 1886–1899. 10.1111/j.0014-3820.2005.tb01059.x 16261727

[B54] Purcell S. Neale B. Todd-Brown K. Thomas L. Ferreira M. A. Bender D.. (2007). PLINK: a tool set for whole-genome association and population-based linkage analyses. Am. J. Hum. Genet. 81, 559–575. 10.1086/519795 17701901 PMC1950838

[B55] Quandt C. A. Bushley K. E. Spatafora J. W. (2015). The genome of the truffle-parasite *Tolypocladium ophioglossoides* and the evolution of antifungal peptaibiotics. BMC Genomics 16:553 10.1186/s12864-015-1777-9 PMC4517408 26215153

[B56] Rhoads A. Au K. F. (2015). PacBio sequencing and its applications. Genomics Proteomics Bioinformatics 13, 278–289. 10.1016/j.gpb.2015.08.002 26542840 PMC4678779

[B57] Roberts R. J. Carneiro M. O. Schatz M. C. (2013). The advantages of SMRT sequencing. Genome Biol. 14:405. 10.1186/gb-2013-14-6-405 23822731 PMC3953343

[B58] Robinson C. H. (2001). Cold adaptation in Arctic and Antarctic fungi. New Phytol. 151, 341–353. 10.1046/j.1469-8137.2001.00177.x

[B59] Rodríguez M. A. Cabrera G. Gozzo F. C. Eberlin M. N. Godeas A. (2011). *Clonostachys rosea* BAFC3874 as a *Sclerotinia sclerotiorum* antagonist: mechanisms involved and potential as a biocontrol agent: *Clonostachys rosea* as a *Sclerotinia sclerotiorum* antagonist. J. Appl. Microbiol. 110, 1177–1186. 10.1111/j.1365-2672.2011.04970.x 21385290

[B60] Rosas A. L. Casadevall A. (1997). Melanization affects susceptibility of *Cryptococcus neoformans* to heat and cold. FEMS Microbiol. Lett. 153, 265–272. 9271852 10.1111/j.1574-6968.1997.tb12584.x

[B61] Ruisi S. Barreca D. Selbmann L. Zucconi L. Onofri S. (2007). Fungi in Antarctica. Rev. Environ. Sci. Biotechnol. 6, 127–141. 10.1007/s11157-006-9107-y

[B62] Saier M. H. Reddy V. S. Tsu B. V. Ahmed M. S. Li C. Moreno-Hagelsieb G. (2016). The Transporter Classification Database (TCDB): recent advances. Nucleic Acids Res. 44, D372–D379. 10.1093/nar/gkv1103 26546518 PMC4702804

[B63] Schadt E. E. Turner S. Kasarskis A. (2010). A window into third-generation sequencing. Hum. Mol. Genet. 19, R227–R240. 10.1093/hmg/ddq416 20858600

[B64] Schmitt M. (1996). The molecular chaperone Hsp78 confers compartment-specific thermotolerance to mitochondria. J. Cell Biol. 134, 1375–1386. 10.1083/jcb.134.6.1375 8830768 PMC2120990

[B65] Schroers H.-J. Samuels G. J. Seifert K. A. Gams W. (1999). Classification of the mycoparasite Gliocladium roseum in Clonostachys as *C. rosea*, its relationship to *Bionectria ochroleuca*, and notes on other Gliocladium-like fungi. Mycologia 91, 365–385. 10.2307/3761383

[B66] Spaniol V. Bernhard S. Aebi C. (2015). *Moraxella catarrhalis* AcrAB-OprM efflux pump contributes to antimicrobial resistance and is enhanced during cold shock response. Antimicrob. Agents Chemother. 59, 1886–1894. 10.1128/AAC.03727-14 25583725 PMC4356836

[B67] Specht C. A. Liu Y. Robbins P. W. Bulawa C. E. Iartchouk N. Winter K. R.. (1996). The chsD and chsE genes of *Aspergillus nidulans* and their roles in chitin synthesis. Fungal Genet. Biol. 20, 153–167. 10.1006/fgbi.1996.0030 8810520

[B68] Stephens M. (2016). False discovery rates: a new deal. Biostatistics. 18, 275–294. 10.1093/biostatistics/kxw041 27756721 PMC5379932

[B69] Storey K. B. Storey J. M. (2012). Insect cold hardiness: metabolic, gene, and protein adaptation. Can. J. Zool. 90, 456–475. 10.1139/z2012-011

[B70] Studholme D. J. Harris B. Le Cocq K. Winsbury R. Perera V. Ryder L.. (2013). Investigating the beneficial traits of *Trichoderma hamatum* GD12 for sustainable agriculture—insights from genomics. Front. Plant Sci. 4:258. 10.3389/fpls.2013.00258 23908658 PMC3726867

[B71] Sun Z. B. Sun M. H. Li S. D. (2015). Draft genome sequence of mycoparasite *Clonostachys rosea* strain 67-1. Genome Announc. 3, e00546–e00515. 10.1128/genomeA.00546-15 26021926 PMC4447911

[B72] Sutton J. C. Li D.-W. Peng G. Yu H. Zhang P. Valdebenito-Sanhueza R. M. (1997). *Gliocladium roseum* a versatile adversary of *Botrytis cinera* in crops. Plant Dis. 81, 316–328. 10.1094/PDIS.1997.81.4.316 30861808

[B73] Syed Ab Rahman S. F. Singh E. Pieterse C. M. J. Schenk P. M. (2018). Emerging microbial biocontrol strategies for plant pathogens. Plant Sci. 267, 102–111. 10.1016/j.plantsci.2017.11.012 29362088

[B74] Talas F. Kalih R. Miedaner T. McDonald B. A. (2016). Genome-wide association study identifies novel candidate genes for aggressiveness, deoxynivalenol production, and azole sensitivity in natural field populations of *Fusarium graminearum*. Mol. Plant Microbe Interact. 29, 417–430. 10.1094/MPMI-09-15-0218-R 26959837

[B75] Tamura K. Stecher G. Peterson D. Filipski A. Kumar S. (2013). MEGA6: molecular evolutionary genetics analysis version 6.0. Mol. Biol. Evol. 30, 2725–2729. 10.1093/molbev/mst197 24132122 PMC3840312

[B76] Teperi E. Keskinen M. Ketoja E. Tahvonen R. (1998). Screening for fungal antagonists of seed-borne *Fusarium culmorum* on wheat using *in vivo* tests. Eur. J. Plant Pathol. 104, 243–251. 10.1023/A:1008647607310

[B77] Treseder K. K. Lennon J. T. (2015). Fungal traits that drive ecosystem dynamics on land. Microbiol. Mol. Biol. Rev. 79, 243–262. 10.1128/MMBR.00001-15 25971588 PMC4429240

[B78] Tzelepis G. Dubey M. Jensen D. F. Karlsson M. (2015). Identifying glycoside hydrolase family 18 genes in the mycoparasitic fungal species *Clonostachys rosea*. Microbiology 161, 1407–1419. 10.1099/mic.0.000096 25881898

[B79] Upchurch R. G. (2008). Fatty acid unsaturation, mobilization, and regulation in the response of plants to stress. Biotechnol. Lett. 30, 967–977. 10.1007/s10529-008-9639-z 18227974

[B80] van Dooremalen C. Suring W. Ellers J. (2011). Fatty acid composition and extreme temperature tolerance following exposure to fluctuating temperatures in a soil arthropod. J. Insect Physiol. 57, 1267–1273. 10.1016/j.jinsphys.2011.05.017 21704631

[B81] Wang K. Li M. Hakonarson H. (2010). ANNOVAR: functional annotation of genetic variants from high-throughput sequencing data. Nucleic Acids Res. 38, e164–e164. 10.1093/nar/gkq603 20601685 PMC2938201

[B82] Wang M. Zheng Q. Shen Q. Guo S. (2013). The critical role of potassium in plant stress response. Int. J. Mol. Sci. 14, 7370–7390. 10.3390/ijms14047370 23549270 PMC3645691

[B83] Wang X. C. Zhao Q. Y. Ma C. L. Zhang Z. H. Cao H. L. Kong Y. M. (2013). Global transcriptome profiles of *Camellia sinensis* during cold acclimation. BMC Genomics 14:415 10.1186/1471-2164-14-415 PMC3701547 23799877

[B84] Wei H. Dhanaraj A. L. Arora R. Rowland L. J. Fu Y. Sun L. (2006). Identification of cold acclimation-responsive rhododendron genes for lipid metabolism, membrane transport and lignin biosynthesis: importance of moderately abundant ESTs in genomic studies. Plant Cell Environ. 29, 558–570. 10.1111/j.1365-3040.2005.01432.x 17080607

[B85] Xie B. B. Qin Q. L. Shi M. Chen L. L. Shu Y. L. Luo Y.. (2014). Comparative genomics provide insights into evolution of trichoderma nutrition style. Genome Biol. Evol. 6, 379–390. 10.1093/gbe/evu018 24482532 PMC3942035

[B86] Xue A. G. Voldeng H. D. Savard M. E. Fedak G. Tian X. Hsiang T. (2009). Biological control of fusarium head blight of wheat with *Clonostachys rosea* strain ACM941. Can. J. Plant Pathol. 31, 169–179. 10.1080/07060660909507590

[B87] Yarden O. Yanofsky C. (1991). Chitin synthase 1 plays a major role in cell wall biogenesis in *Neurospora crassa*. Genes Dev. 5, 2420–2430. 1836444 10.1101/gad.5.12b.2420

[B88] Zhang G. Storey J. M. Storey K. B. (2018). Elevated chaperone proteins are a feature of winter freeze avoidance by larvae of the goldenrod gall moth, *Epiblema scudderiana*. J. Insect Physiol. 106, 106–113. 10.1016/j.jinsphys.2017.04.007 28433751

[B89] Zhu W. Zhan J. McDonald B. A. (2018). Evidence for local adaptation and pleiotropic effects associated with melanization in a plant pathogenic fungus. Fungal Genet. Biol. 115, 33–40. 10.1016/j.fgb.2018.04.002 29626634

