# Supplementary figures and images for: Out in the Cold: Identification of Genomic Regions Associated With Cold Tolerance in the Biocontrol Fungus Clonostachys rosea Through Genome-Wide Association Mapping
Source: Front Microbiol. 2018 Nov 22;9:2844. doi: 10.3389/fmicb.2018.02844 (PMC6262169; doi:10.3389/fmicb.2018.02844)

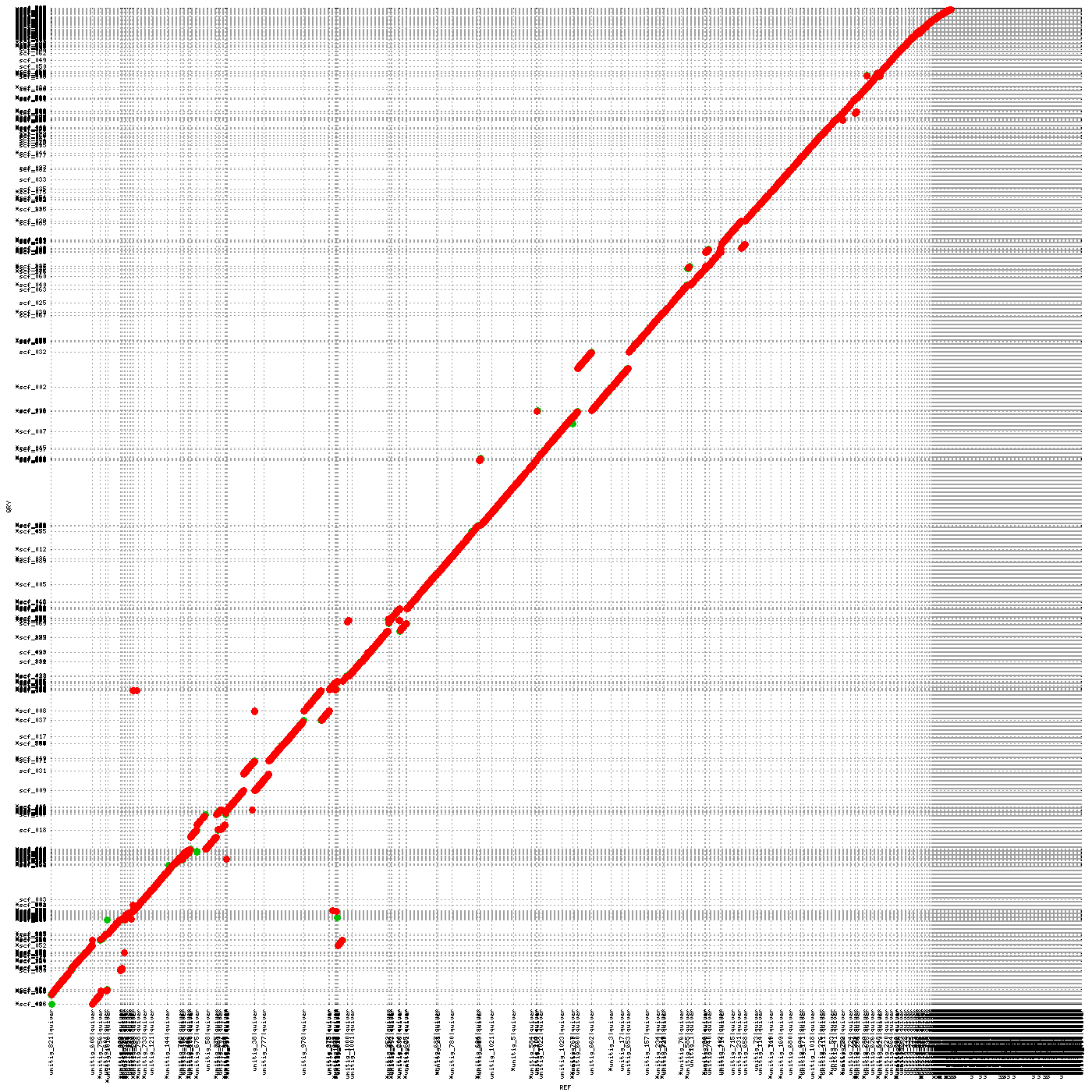

Supplement: Supplementary Figure 1 — Origin of strains does not correlate with growth on PDA at 10°C. Using principal component analysis, we compared growth rates on PDA at 10°C, PDA at room temperature, and adjusted growth at 10°C to room temperature. The analysis revealed no correlation to the strains' origin of isolation. [file Image_1.PNG]

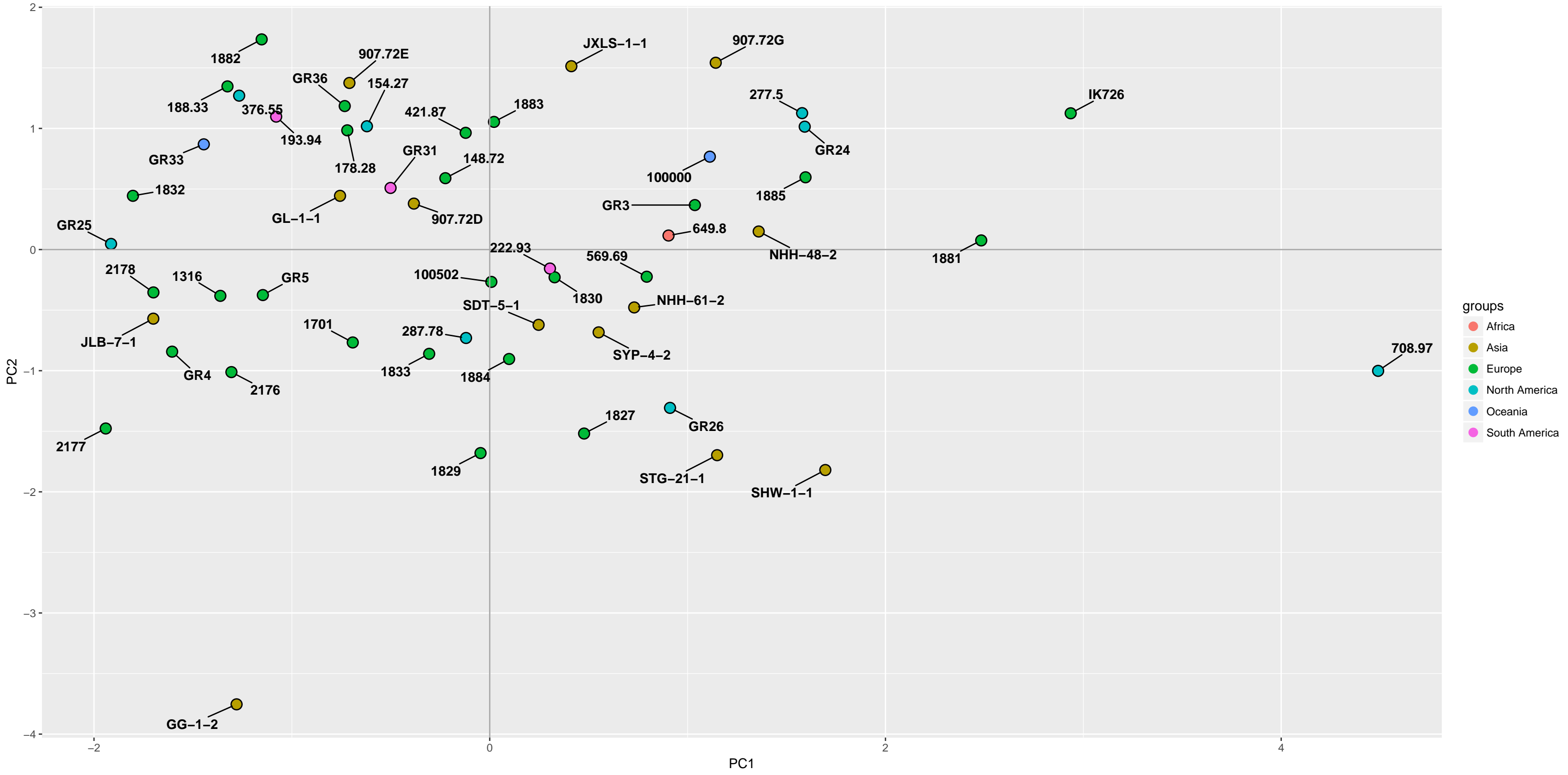

Supplement: Supplementary Figure 2 — MUMmer alignment output of PacBio long contigs to Clonostachys rosea IK726 ver. 1 genome scaffolds. [file Image_2.pdf]
